# A comparison between different variants of the spatial Stroop task: The influence of analytic flexibility on Stroop effect estimates and reliability

**DOI:** 10.3758/s13428-023-02091-8

**Published:** 2023-03-09

**Authors:** Giada Viviani, Antonino Visalli, Livio Finos, Antonino Vallesi, Ettore Ambrosini

**Affiliations:** 1 Department of Neuroscience, University of Padova, 35121 Padova, Italy; 2 Padova Neuroscience Center, University of Padova, Padova, Italy; 3 Department of Developmental Psychology and Socialization, University of Padova, Padova, Italy; 4 Department of General Psychology, University of Padova, Padova, Italy

**Keywords:** Stroop task, Spatial Stroop, Reliability, Analytical flexibility, Multilevel modelling

## Abstract

The spatial Stroop task measures the ability to resolve interference between relevant and irrelevant spatial information. We recently proposed a four-choice spatial Stroop task that ensures methodological advantages over the original color-word verbal Stroop task, requiring participants to indicate the direction of an arrow while ignoring its position in one of the screen corners. However, its peripheral spatial arrangement might represent a methodological weakness and could introduce experimental confounds. Thus, aiming at improving our “Peripheral” spatial Stroop, we designed and made available five novel spatial Stroop tasks (Perifoveal, Navon, Figure-Ground, Flanker, and Saliency), wherein the stimuli appeared at the center of the screen. In a within-subjects online study, we compared the six versions to identify which task produced the largest but also the most reliable and robust Stroop effect. Indeed, although internal reliability is frequently overlooked, its estimate is fundamental, also in light of the recently proposed reliability paradox. Data analyses were performed using both the classical general linear model analytical approach and two multilevel modelling approaches (linear mixed models and random coefficient analysis), which specifically served for more accurately estimating the Stroop effect by explaining intra-subject, trial-by-trial variability. We then assessed our results based on their robustness to such analytic flexibility. Overall, our results indicate that the Perifoveal spatial Stroop is the best alternative task for its statistical properties and methodological advantages. Interestingly, our results also indicate that the Peripheral and Perifoveal Stroop effects were not only the largest, but also those with highest and most robust internal reliability.

## Introduction

The Stroop task (Stroop, 1935) is one of the most seminal behavioral paradigms in experimental psychology. It is commonly used to investigate cognitive control (i.e., the ability to regulate thoughts and actions according to behavioral goals; Braver, 2012) and, specifically, interference resolution (Nee et al., 2007). In the original and most widely used version of this task, referred to as the color-word Stroop task, participants are asked to name the ink color of words denoting color names. This task allows researchers to explore the resolution of interference produced on incongruent trials (e.g., MacLeod, 1991; Stroop, 1935): it takes longer (and attracts more errors) to name the ink color of a word that denotes a different color name (incongruent trials) as compared to when the ink color and the word meaning match (congruent trials), the so-called Stroop effect^1^. According to multiple-stage (e.g., De Houwer, 2003; Zhang & Kornblum, 1998) and multiple loci (e.g., Augustinova et al., 2019; Parris et al., 2022) accounts of Stroop interference, the Stroop effect is produced because in incongruent trials participants are required to overcome the interference or conflict at the task, stimulus and response levels. There is indeed an interference between two competing processing streams, reading and color naming, with the former more prevailing than the latter, between relevant and irrelevant stimulus dimensions, and also between the different vocal responses activated by the ink color and the color name (De Houwer, 2003; Freund et al., 2021; Funes et al., 2010).

Despite its widespread and long-standing use, an overlooked and debated methodological aspect of the verbal Stroop task is that it requires vocal responses to fully exert its interference at both the stimulus and the response level (see e.g., MacLeod, 1991). Indeed, the Stroop task is strongly dependent on response modality, as delineated by the dimensional overlap model put forward by Kornblum (1992), which outlines the requirements that need to be satisfied to consider a task as a Stroop task. Indeed, while color-word Stroop tasks requiring vocal responses are categorized as a “type-eight ensemble” (i.e., a Stroop task according to Kornblum’s classification), ensuring dimensional overlap not only between relevant and irrelevant stimulus dimensions but also between them and the response, those requiring manual responses are categorized as a “type-four ensemble” (i.e., a Stroop-like task according to Kornblum’s classification) because they lack the overlap between stimulus and response dimensions. Accordingly, the color-word Stroop task has been shown to produce larger Stroop effects with vocal as compared to manual responses (e.g., Augustinova et al., 2019).

Several adaptations of the original color-word Stroop task have been proposed in the literature, including spatial versions investigating the interference between relevant and irrelevant spatial information. Among these, we recently designed a spatial Stroop task in which participants are asked to attend to and indicate the direction of an arrow (i.e., the task-relevant feature) while ignoring the location in which it appears (i.e., the task-irrelevant feature) (Puccioni & Vallesi, 2012a, 2012b). Our spatial version of the Stroop task works exactly as the verbal Stroop task and can be considered as one of its purely spatial variants (for a more detailed discussion, see Viviani et al., 2022). Indeed, it ensures a multiple-loci interference at the task, stimulus and response levels. First, it entails an asymmetric relation between the stimulus dimensions because the position of a visual stimulus can be assumed to be processed in a preponderant way as compared to other visual characteristics, such as the pointing direction in the case of an arrow (Lu & Proctor, 1995), thus engendering conflict between two competing tasks. Moreover, it yields conflict both at the stimulus and response levels as it is a “type-eight ensemble” (Kornblum, 1992) because the arrows appear in one of the four corners of the screen and point in one of the same four directions (i.e., upper-left, upper-right, lower-right, and lower-left), thus ensuring the dimensional overlap between the relevant and irrelevant characteristics of the stimulus, and participants provide their responses by using four keys that are spatially arranged to ensure the dimensional overlap between the stimulus and response dimensions. Therefore, our spatial Stroop task assesses the same central interference resolution processes as verbal Stroop tasks. Furthermore, mouse responses can also be employed, thus allowing researchers to investigate the temporal dynamics of the interference resolution processes (Tafuro et al., 2020).

Our spatial Stroop task also presents several methodological advantages over the original, verbal Stroop task. First, it allows to exclude linguistic processing, which might be beneficial, for example, when examining cognitive control in populations with language or reading disorders, as the performance would not be negatively affected by the impaired linguistic abilities. Second, the use of spatial stimuli to investigate cognitive control might promote a more domain-general understanding of cognitive control mechanisms, overcoming the prevalence of linguistic-based accounts and allowing to better investigate hemispheric lateralization by minimizing a potential confound represented by task verbal demands (Ambrosini & Vallesi, 2017; Tafuro et al., 2019). Third, as discussed above, the verbal Stroop task requires vocal responses to fully exert its interference, but they present some methodological issues. Indeed, the current gold standard to assess verbal response times (RTs) is still to mostly rely on human coding, which is extremely tedious, time-consuming, and prone to errors, biases, and other sources of measurement error. Moreover, it is currently not very feasible to reliably record vocal responses in online studies. Finally, vocal responses are problematic for neuroimaging studies, as they introduce movement artifacts related to overt speech that can contaminate the cognitive control-related signals of interest, also considering that they are temporally non-random, but covary with signal of interest because they are somewhat time-locked to the task timing. By contrast, the spatial Stroop task allows using manual responses, which are easier to record with minimal motion artifacts and low measurement error even in online studies.

Moreover, our spatial Stroop task allows overcoming some methodological issues that are present in most of the spatial versions of the Stroop tasks used in the literature, as we recently discussed in detail (Viviani et al., 2022). Indeed, position-word tasks (with location words displayed in congruent or incongruent positions; e.g., White, 1969) and arrow-word tasks (with location words embedded or flanked by congruent or incongruent arrows; e.g., Shor, 1970) had often been employed, but both still rely on verbal processing and do not ensure a complete spatial Stroop effect due to the lack of a full dimensional overlap between stimulus and response dimensions. Moreover, most studies (including those employing purely spatial Stroop tasks, e.g., Funes et al., 2007; Pires et al., 2018) used two-alternative forced-choice tasks, but this prevents having complete repetition-free trial sequences (for which at least four options are needed), thus making it hard to distinguish conflict resolution/adaptation effects from low-level binding, positive and negative priming effects (for a detailed discussion, see Viviani et al., 2022 and Puccioni & Vallesi, 2012b).

Notwithstanding its advantages, our spatial Stroop task also presents some methodological limitations. Indeed, the arrows appear in peripheral locations on the screen, therefore requiring the deployment of large visuospatial attention shifts and eye movements. This can affect the behavioral measures of the interference resolution processes of interest, especially for extreme visual eccentricities and when mouse movements are required. Moreover, this can also introduce oculomotor artifacts that, albeit not being as problematic as the overt speech and motor artifacts produced by vocal responses, can still contaminate the cognitive control-related signals of interest in neuroimaging studies.

Our aim is to propose novel spatial Stroop tasks that allow overcoming these issues of our original spatial Stroop task, which we will call “Peripheral” due to its spatial arrangement with high stimulus eccentricity. To this aim, we designed five alternative versions allowing for presentation of the experimental stimuli at the center of the screen, while maintaining all the required methodological criteria to yield a complete spatial Stroop interference effect and a complete alternation of the trial sequences. One of these versions, the “Perifoveal” one, is a direct adaptation of the Peripheral version in which the arrows appear in perifoveal locations. The other four alternative versions were inspired by the other experimental paradigms generating interference (e.g., the Flanker task) that allowed preserving both the fundamental characteristics of a spatial Stroop task and its methodological advantages over the original verbal Stroop task, as described above. It is noteworthy that these four alternative versions differed from the Peripheral and Perifoveal ones in the manipulation used to engender conflict at the task level. Indeed, to generate asymmetry between the two dimensions, we leveraged not only the higher processing automaticity of one dimension as compared to the other, but also the higher discriminability/perceptual salience of one dimension relatively to the other, and these two manipulation types were present in different amounts in the newly created tasks. A detailed description of them will be provided in the Methods section.

These alternative versions were evaluated in terms of two important features. The first one is their ability to show a large Stroop effect for manual RTs, in line with the dominance assumption (Rouder & Haaf, 2018, 2019): all individuals truly respond slower in incongruent than congruent trials (or, in other words, nobody should show a non-positive Stroop effect). The second one is the internal reliability of their Stroop effects. Indeed, as pointed out by Hedge and coworkers (2018a), the reliability of an experimental effect represents a frequently overlooked statistical issue that, if not taken into account properly, might jeopardize the effectiveness of correlational research in cognitive neuroscience and psychology (e.g., Dang et al., 2020; Elliott et al., 2020; Rouder et al., 2019; Wennerhold & Friese, 2020).

Finally, population-level effects and internal reliability were evaluated under different analytical frameworks. The reason is that both cross-subject and cross-trial variability influence estimates of population-level effects and their reliability (Chen et al., 2021). However, the classical test theory analytical approach requires computing participants-by-task scores by averaging participants’ performance across trials. This discards any cross-trial variability that may contaminate participants-by-task scores, potentially decreasing not only their accuracy and generalizability, but also their reliability (see also Rouder & Haaf, 2019). Therefore, we used not only a classical analytical approach, but also a multilevel modelling approach that allows assessing the experimental effects of interest while removing intra-subject, trial-by-trial noise, and effects of lower-level confounding factors.

## Methods

We report how we determined our sample size, all data exclusions, all inclusion/exclusion criteria, all manipulations, and all measures in the study. All inclusion/exclusion criteria were established prior to data analysis. All data and materials are available from our project repository on the Open Science Framework (OSF) platform at osf.io/5sm9j. No part of the study, including the analyses, was pre-registered.

## Procedure and materials

Participants were administered with six versions of a four-choice spatial Stroop task, all requiring keypress responses to indicate the direction of a target arrow. The experiment was programmed using Psytoolkit (Stoet, 2010, 2017) and administered online (the code and stimuli are available on OSF: osf.io/9hsnw). All the participants were recruited by the experimenters and given a link to perform the task online.

**Fig. 1:**
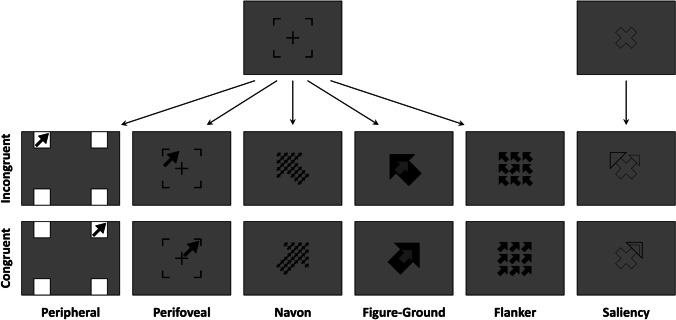
Experimental stimuli for the six spatial Stroop tasks. For each version, an example of incongruent and congruent trials is depicted. The *first row* shows the fixation stimuli. Note that the stimuli are not to scale for illustrative purposes (see Procedure and Materials for details)

The stimuli were presented in full-screen mode, with a resolution of 800 x 600 pixels, on a grey background (RGB: 128, 128, 128). Each trial started with a fixation stimulus presented at the center of the screen for 500 ms, which participants were instructed to fixate. For all but the Saliency Stroop task, the fixation stimulus consisted in a vertically oriented thin black cross (30 x 30 pixels) enclosed in the partial outline of a black square (94 x 94 pixels), which was then replaced by the experimental stimulus (see Fig. 1). Moreover, in the Peripheral Stroop task, four white squares (73 x 73 pixels) were also presented during the fixation screen at the four corners of an imaginary square of 600 x 600 pixels centered on the screen. In the Saliency Stroop task, the fixation stimulus consisted in the black outline of a thick diagonal cross (60 x 60 pixels, see Fig. 1). Then, the experimental stimulus was presented, which was different for each experimental task (see below). The stimulus remained on screen until participant’s response or up to a response time-out of 2000 ms. Afterwards, a blank screen constituting the inter-trial interval was presented for 500 ms.

Each of the six spatial Stroop tasks followed the same procedure outlined above but involved different experimental stimuli, as detailed below. However, in all the tasks, participants had to pay attention to a target arrow to indicate its direction, which was thus the task-relevant information, corresponding to the response. The possible directions of the target arrows were always upper-left, upper-right, lower-right, and lower-left and participants were required to provide their responses by using four keys on a computer keyboard, which were spatially arranged to ensure the dimensional overlap between the characteristics of the stimulus and the response. Indeed, the keys E, O, K, and D were associated, in a spatially compatible fashion, with the upper-left, upper-right, lower-right, and lower-left direction, respectively, and had to be pressed using the left middle, right middle, right index, and left index fingers, respectively. The experimental stimuli also included a task-irrelevant feature that could either match or not with the task-relevant feature, respectively, in congruent and incongruent trials, thus interfering with the participants’ decision in incongruent trials (see Fig. 1):

1. in the *Peripheral Stroop*, the experimental stimulus consisted in an arrow that appeared inside one of the four white squares, pointing to one of the same four directions (upper-left, upper-right, lower-right, and lower-left). Participants were instructed to respond to the arrow direction, regardless of the position where it appeared. Trials could thus be either congruent or incongruent, depending on whether the arrow direction (i.e., the task-relevant information) matched or not its position (i.e., the task-irrelevant information);
2. The *Perifoveal Stroop* version was akin to the *Peripheral* one with the difference that the arrow position was manipulated inside the fixation stimulus. Indeed, the partial outline of the square around the fixation cross created the impression of four small squares. Therefore, the arrow was displayed within one of these apparent small squares and, like in the *Peripheral Stroop task*, its pointing direction (i.e., the task-relevant information) could be either congruent or incongruent with its position (i.e., the task-irrelevant information).
3. In the *Navon Stroop* version, the experimental stimulus consisted of 28 small arrows (local level), spatially arranged to form a large arrow (global level). Participants were asked to indicate the direction of the small arrows (i.e., the task-relevant information), regardless of the direction of the large arrow (i.e., the task-irrelevant information). All the small arrows pointed to the same direction which, to manipulate congruency, either matched or not the direction of the large arrow. This task represents a modification of the original Navon task (Navon, 1977) assessing letter identification, whose typical effect relies on the so-called global precedence. In a similar vein, our spatial version using arrows also entailed task-related interference between the more salient and also conceptually stronger processing of the global arrow as compared to the local arrows. Moreover, stimulus conflict was guaranteed by the dimensional overlap between the task-relevant and task-irrelevant arrows, while response conflict was produced by the two dimensional overlaps between response and stimulus information.
4. In the *Figure-Ground Stroop* version, the experimental stimulus consisted of a small grey arrow completely embedded in a large black arrow. The task-relevant information was the direction of the smaller arrow and the task-irrelevant information was the direction of the outer arrow, and the two arrows could either match or not. Like the Navon Stroop versions, also this task leverages global precedence, with easier processing of the large arrow compared to the small one, with a consequent global-to-local conflict engendering task-related interference. Moreover, interference was produced at the stimulus level thanks to the dimensional overlap between the two arrows and at the response level due to the stimulus-response dimensional overlaps.
5. In the *Flanker Stroop,* the experimental stimulus consisted of a black arrow presented in the center of the screen (target), flanked by 8 same-sized black arrows on a 3-by-3 square grid. The task-relevant information was the direction of the central arrow, which could be congruent or not with the flanking arrows. This task represents a modification of the original Flanker task (Eriksen & Eriksen, 1974) assessing letter identification, whose typical interference effect is driven by the higher perceptual numerosity of flanking stimuli as compared to the centrally presented target. Similarly, our spatial version with arrows leveraged the higher perceptual saliency due to numerosity of the surrounding task-irrelevant arrows as compared to the central task-relevant arrow, producing task conflict. Moreover, interference at the stimulus level was caused by conceptual overlap^2^ between the flanking distractors and the central arrow, while interference at the response level was generated by the two dimensional overlaps between the response and the stimulus information.
6. The *Saliency Stroop* version slightly differed from the others, starting with a different fixation cross, which was not replaced by the experimental stimulus. Indeed, the experimental stimulus consisted in two empty triangles, a smaller and thinner one and a larger and thicker one, which appeared at the extremity of one of the four cross arms. In incongruent trials, the two triangles appeared at different extremities, forming an arrow with a large head and one with a small head (target arrow) pointing to different directions; in congruent trials, the two triangles appeared at the same extremity, creating a single arrow with overlapping heads. Participants responded to the direction of the smaller arrow, regardless of the direction of the larger arrow, which was perceptually more salient and, thus, processed more easily. Therefore, in this task, conflict was exclusively driven by this saliency imbalance between the two dimensions. Similarly to the other tasks, stimulus-related conflict occurred due to stimulus-stimulus overlap, while response-related conflict was driven by the two-dimensional overlaps between response and stimulus information.

At the beginning of the experiment, general instructions were provided, informing participants of the procedure, the general task (i.e., indicating the direction of a target arrow), and the response mapping. It was also recommended to execute the experiment in a quiet environment without distractions and to maintain a comfortable posture that allowed them to look straight to the center of the screen and keep the responding fingers in contact with the response keys. Particular care was taken to keep the instructions as simple and clear as possible.

The six tasks were then presented in separate blocks of 72 experimental trials each, preceded by one warm-up trial that was not included in the analyses. The experimental trials were equally divided in congruent and incongruent trials and both the four task-irrelevant and task-relevant characteristics (and thus the responses) were presented the same number of times. A self-paced break was provided between the blocks, during which a reminder of the response mapping was presented (median duration = 28.9 s, *IQR* = 23.0 s). The experiment lasted less than 23 min in total on average (M = 22.7 min, SD = 7.1 min).

At the beginning of each block, specific task instructions were presented, illustrating the stimuli and the task to be performed. Participants were also asked to respond as quickly and accurately as possible. The instructions were followed by a sub-block of practice trials, during which participants received feedback on their responses. In case of errors, the task-relevant information and the corresponding response mapping were repeated. Practice trials were presented until participants reached an accuracy of 75% within at least six trials, or for a maximum of 24 trials. In two cases (0.46% of all cases), the accuracy was below 75% within 24 trials, but it was still well above the chance level (62.5 and 70.8%); in four other cases (0.93% of all cases) more than six trials were needed to reach the required level of accuracy.

A randomized balanced order of presentation of the six tasks was used, based on a Williams Latin square design (Williams, 1949) to also account for first-order carryover effects. The order of presentation of the trials was pseudo-randomized so that there were at most three consecutive repetitions of congruency, and no repetitions of stimulus characteristics and/or responses, thus avoiding first order priming effects.

### Data analysis

The data were analyzed in Jamovi (version 1.6; jamovi.org) and Matlab (version 2019b; The MathWorks, Inc. Natick, MA) to 1) estimate the magnitude of the Stroop effects in the six tasks we used, as well as their across-tasks differences, and 2) estimate their internal consistency reliability based on two analytical approaches. Analyses were performed not only on untransformed RTs, but also on both natural log-transformed RTs (lnRTs) and inverse-transformed RTs (iRTs, computed as -1000/RTs). Indeed, the distribution of RTs was heavily right-skewed and their logarithmic transformation did not eliminate completely the right skewness, so that the lognormal distribution did not provide an adequate fit to our data (see Supplementary Materials at osf.io/djszu, Section 1).

We first performed a “standard” general linear model (GLM) analysis, commonly used in cognitive psychology research, to facilitate comparison of our results with existing (and future) findings. This analytical approach requires to aggregate participants’ performance across trials to obtain participants-by-task scores, in line with the analytic framework of the classical test theory. However, this discards any trial-by-trial variability that may contaminate participants-by-task scores, potentially decreasing their accuracy and generalizability (see also Rouder & Haaf, 2019).

To avoid this potential problem, we used a multilevel modeling approach (or trial-by-trials hierarchical modeling), which is particularly useful to our aim. Indeed, multilevel modeling allows assessing the experimental effects of interest (i.e., in our case, the Stroop effects in the different tasks and the differences between them) while partialing out the effects of lower-level confounding factors at the trial level, which can be seen as sources of trial-by-trial noise in the estimation of the Stroop effects at the subject level. This approach would thus provide more accurate and precise estimates of Stroop effects and, consequently, of their reliability as well, by explaining intra-subject/inter-trial sources of variance contributing to measurement error.

Moreover, we assessed the robustness of our results to analytic flexibility (the so-called “researcher degrees of freedom” (Simmons et al., 2011) or “the garden of forking paths” (Gelman & Loken, 2013) by performing a series of robustness checks based on a multiverse analysis approach (Parsons, 2020; Steegen et al., 2016).

Training trials, trials with errors or missed responses (3.97% of trials) and trials with RTs shorter than 150 ms (none of the remaining trials) were excluded from all the analyses. We checked for the presence of participants with low compliance, defined as those having either a mean iRTs more than three standard deviations away from the sample mean or a mean accuracy lower than 75%. Based on these criteria, no participant was excluded from the analyses (see osf.io/djszu, Section 2).

#### Assessing the magnitude of the Stroop effects

We first performed a standard GLM analysis using a repeated-measures ANOVA (rmANOVA) with Task (Peripheral, Perifoveal, Navon, Figure-Ground, Flanker, and Saliency) and trial Congruency (Congruent vs. Incongruent) as within-subject factors, and the Huynh-Feldt correction for sphericity violations were applied to the degrees of freedom. Post-hoc comparisons were performed using paired *t* tests corrected for multiple comparisons with the Scheffé’s method. To better investigate the Task by Congruency interaction of interest (i.e., the across-tasks differences in the Stroop effect), a follow-up rmANOVA was performed on the Stroop effects (i.e., the difference between RTs in Incongruent and Congruent trials) across the six tasks. The statistical significance of the Stroop effect for each task was assessed by means of two-tailed one-sample *t* tests against 0 and the corresponding effect size estimates were computed as the Cohen’s *d*. These analyses were performed on both RT and accuracy measures (for which we also performed a nonparametric Friedman ANOVA on Stroop effects).

We then performed two multilevel modelling analyses, that is, a linear mixed-effect model analysis (LMM, Baayen et al., 2008) and a random coefficient analysis (RCA, also called random regression or two-step regression, Lorch & Myers, 1990). We also performed RCA because LMMs usually fail to converge when trying to model complex random effects structures, that is, the inter-subject variability in experimental (and confounding) effects, especially when random effects are large and/or there are few observations (Barr et al., 2013). Consequently, usually a simpler random-effects structure has to be modeled, thus constraining the remaining effects to be the same across participants. This assumption, however, is often untenable, severely limits the generalizability of the results, and prevents assessing the reliability of the effects for which the inter-subject variability is not modeled. By contrast, RCA allows assessing the inter-subject variability in all the modeled effects. Indeed, in RCA, by-subject regressions are first computed, thus allowing all the modeled effects to vary across participants, followed by a one-sample *t* test against zero (or equivalent nonparametric tests) to test for their statistical significance.

We included in the LMM several possible confounding predictors that were expected to explain trial-by-trial variability in RTs. Indeed, as explained above, the aim was to obtain more accurate estimates of the Stroop effects, which can indeed be contaminated by these sources of removable noise at the trial level, like longitudinal effects during the task. Specifically, the final model for LMM comprised the following lower-level confounding predictors as fixed effects: i) three continuous predictors for the effect of both the rank-order of the blocks in the experiment (Block) and the rank-order of the trials in each block (Trial), as well as their interaction, to account for potential time-on-task effects like learning/adaptation or fatigue effects (e.g., Möckel et al., 2015); ii) a continuous predictor reflecting the RT of the preceding trial (preRT), to account for the well-known temporal dependency in response times (Baayen & Milin, 2010); iii) a predictor for the fixed effect of error commission in the preceding trial (PostERR), to account for the so-called post-error slowing (Rabbitt, 1966). It is important to note that these fixed effects were modeled not only to improve the model fit and the estimation of the effects of interest, but also to avoid violating the assumption of the independence of observation for linear modeling. We also included two other lower-level confounding predictors as fixed effects, that is, the horizontal and vertical coding of the response (respectively, hResp and vResp), to account for potential differences due to the response hand and finger, respectively. Finally, we modeled the experimental effects of interest and their inter-individual variability by including predictors for the effects of the Stroop version (Task), the trial congruency (Cong), and their interaction, which were included in both the fixed and random parts of the model (the inclusion of the effects of interest in the random part is necessary for calculating their reliability). The Wilkinson-notation formula for the final model is:

$$\kern0.5em RT\sim 1+ Block\ast Trial+ preRT+ PostERR+ hResp+ vResp+ Task\ast Cong+\left( Task\ast Cong| Participant\right)$$

The continuous predictors were scaled to facilitate model convergence and the interpretation of the results. We determined the final LMM model by performing a model-building procedure to assess whether the inclusion of the parameters for the above-mentioned effects was justified, using a log-likelihood ratio test to compare progressively more complex models with simpler models (Baayen et al., 2008). After this model-building procedure, we inspected the quantile-quantile plot for the residuals of the final model for evidence of stress in the model fit. We then refitted the final model after removing data points with absolute standardized residuals exceeding 3. We report the estimated coefficient (*b*), standard error (*SE*), and *t* and *p* values for each fixed effect included in the trimmed final model (note that all the results reported in the present paper refer to the trimmed version of the final models). We calculated the *p* values by using Satterthwaite's approximation of degrees of freedom. An alpha level of .05 was set as the cut-off for statistical significance. Effect sizes for the Task by Congruency interaction of interest were estimated as Cohen’s d based on one-sample *t* tests on the random slopes.

As regards the RCA, linear regressions were first run at the subject level using a regression model similar to the final LMM model described above. In this case, the Block predictor was not included in the model because it was confounded with the Task predictor at the subject level. The corresponding formula for the final RCA model is thus:

$$RT\sim1+Trial+preRT+\;PostError+hResp+vResp+Task\ast Cong$$

Again, the model was refitted after exclusion of outliers as described above. Then, the statistical significance and the effect size of the modeled effects was assessed at the group level by performing one-sample *t* tests against 0 on the estimated *b* coefficients for each participant.

We also performed control analyses using multilevel modelling to verify the assumption that this analytical approach provides better estimates of Stroop effects and their reliability by explaining intra-subject/inter-trial sources of variance contributing to measurement error. To this aim, we replicated both LMM and RCA analyses on iRTs without the inclusion of all the trial-level confounds described above and compared their results with those yielded by the full models.

Finally, we examined commonality among spatial Stroop tasks by performing a correlational analysis. Specifically, we computed Pearson's correlations among the participants’ Stroop effects for the six tasks, separately for the GLM, LMM, and RCA analyses. The statistical significance of these correlations was corrected for multiple comparisons using the FDR method. We also performed an exploratory factor analysis on the participants’ Stroop effects using the maximum likelihood extraction method followed by an Oblimin rotation. The number of factors to be extracted was determined based on parallel analysis.

### Assessing the internal reliability of the Stroop effects

We first assessed the internal consistency reliability of the GLM-based aggregated Stroop effect in each task by computing split-half correlations corrected with the Spearman–Brown formula (*r*_*SB*_). Specifically, for each task, the observations were randomly split in two subsets and the participants’ Stroop effects were computed and correlated between the two subsets.

We then assessed the internal reliability of the Stroop effects estimated using the multilevel modelling approach. Specifically, for each task, the observations were randomly split in two subsets and both LMM and RCA analyses were performed for each subset to model the inter-individual variability in the Stroop effect while controlling for the same confounding predictors used in the main analyses described above. Finally, for both the LMM and RCA analyses, the by-subject random slopes for the Cong effects for each task in the two subsets were correlated to obtain the *r*_*SB*_ values. We also computed the reliabilities of the Stroop effects yielded by the reduced multilevel models described above, which did not include trial-level confounds.

In all cases, 2000 randomizations were used. We report the median *r*_*SB*_ values as well as the corresponding nonparametric 95% confidence intervals (*CI*_95%_).

## Participants

Seventy-two participants were recruited (42 females and 27 males; mean age = 25.35 years, SD = 8.21 years; three participants choose to not indicate gender and age). Participants consisted of a convenience sample recruited using researchers’ personal networks and were not compensated for their participation.

Participants’ handedness was assessed using the Edinburgh Handedness Inventory (EHI, Oldfield, 1971). The sample comprised six left-handed participants (EHI scores < −50) and nine ambidextrous participants (EHI score between −50 and 50), but the results were substantially the same when excluding either left-handed participants only or together with ambidextrous participants. Two participants reported suffering from neurological or psychiatric disorders and to be under medication. Again, the exclusion of these participants did not substantially change the reported results, so we decided to not exclude them (see the “LMM - iRT - Control analyses” section of the analysis script available at osf.io/9xfkw).

### Power analysis

We performed an a-priori power analysis in G*Power (Erdfelder et al., 1996) to compute the minimum sample size required to detect, with a statistical power of .80, the interaction of main interest (i.e., the difference in the Stroop effect across tasks) in a repeated measure ANOVA. We assumed a small-medium Cohen’s *d* effect size of .3 (corresponding to *η*^2^_p_ = .022), a correlation between repeated measures of .70, and a (Huynh-Feldt) non-sphericity correction *ε* of .5, as estimated conservatively from recent pilot studies with a similar design from our research group. This analysis revealed that at least 48 participants were required. We nonetheless decided to recruit as many participants as possible exceeding the required sample size, so as to be able to detect even smaller effects (by increasing the statistical power of our analyses) and to increase the precision of the experimental effects estimates.

As regards the power estimation for the LMM analysis, it “is still a largely uncharted terrain containing many open and unresolved issues” (Kumle et al., 2021, p. 3). First, classical analytical approaches to power estimation, like the one used by G*Power, cannot be applied to LMMs because they lack the required flexibility (Kumle et al., 2021). Moreover, to the best of our knowledge, the available analytical solutions proposed to compute power for LMMs are not adequate for our complex model (e.g., the Westfall’s approach (Westfall et al., 2014) is only applicable for models with a single two-level fixed effect; see Brysbaert & Stevens, 2018). To solve these issues, the simulation-based approach to power analysis has been proposed for LMMs as a flexible and powerful alternative to analytical approaches (Brysbaert & Stevens, 2018; Green & MacLeod, 2016; Kumle et al., 2021). However, this approach is not suitable in our case, as it requires that an optimal model is selected a-priori, while we adopted a conservative hierarchical model building approach. Moreover, given our aim and the use of novel experimental tasks, no well-powered data are available to allow us to generate accurate artificial data needed to run the simulation, and the complexity of our model and the amount of recorded data made it too computationally intensive to run multiple simulations with varying parameters. Nonetheless, it should be noted that, since we had 36 trials per condition, 45 participants were needed to reach the recommended minimum number of 1600 observations per condition (Brysbaert & Stevens, 2018). It should also be noted that LMMs tend to provide higher power than standard GLM approaches.

## Results

### Magnitude of the Stroop effects

Table 1 shows the descriptive statistics for the RTs (see osf.io/djszu, Section 3, for the other measures) and the accuracy (percentage of correct trials). Participants’ overall accuracy was very high (M = 96.0%, range = [86.3–99.8%]) and at ceiling in all tasks for congruent trials (>98.3%; M = 98.7%, range = [93.5–100%]) but not for incongruent trials (>89.5%; M = 93.3%, range = [77.3–100%]). Consequently, the participants’ Stroop effects on the accuracy heavily depended on their average accuracy (i.e., participants with a perfect overall accuracy cannot show a Stroop effect). This severely limits the interpretability of the analyses on accuracy and introduces strong biases in the estimation of the reliability of this measure. For this reason, we do not report here the results of the analyses on the accuracy (but see osf.io/6rzsh for the GLM analysis on accuracy data) and did not assess the internal reliability of this measure.

**Table 1:** Descriptive statistics

	Peripheral		Perifoveal		Navon		Figure-Ground		Flanker		Saliency	
	*M*	*SD*	*M*	*SD*	*M*	*SD*	*M*	*SD*	*M*	*SD*	*M*	*SD*
*Accuracy*												
C	99%	3%	98%	4%	99%	2%	99%	2%	99%	2%	98%	2%
I	90%	9%	90%	11%	96%	6%	94%	8%	95%	7%	96%	6%
Stroop	9%	8%	8%	9%	3%	6%	5%	8%	4%	7%	3%	5%
*RT (ms)﻿*												
C	586	158	529	169	544	114	500	90	523	124	490	105
I	716	214	658	219	615	140	579	90	586	108	523	108
Stroop	130	87	129	78	71	47	79	29	63	31	32	28

C, congruent trials; I, incongruent trials

We report here the results of the analyses performed on iRTs because the distribution of RTs was heavily right-skewed (see Data analysis section). Moreover, the assumption of normality was violated for the residual of the analyses on both lnRTs and RTs, and the latter also severely violated the homoscedasticity assumption (see osf.io/djszu, Sections 5.2.2. 5.3.2, 6.2.2, and 6.3.2). However, the results of the analyses performed on RTs and lnRTs are reported in the supplementary material available from our project repository on the Open Science Framework (see osf.io/djszu, Sections 4.2. 4.3, 6.2, and 6.3).

**Fig. 2:**
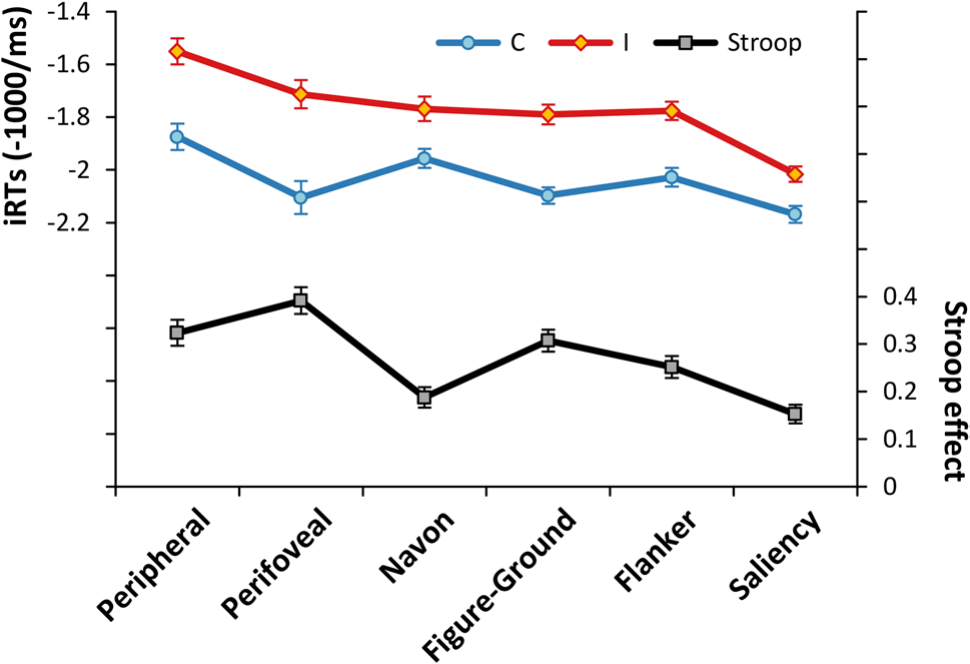
ANOVA results, Congruency by Task interaction. The plot shows the participants’ mean iRTs in Congruent (C, *blue line*) and Incongruent (I, *red line*) trials as a function of the Task (*x-axis*). The corresponding Stroop effects are also shown (*black line*). *Error bars* represent within-subjects 95% confidence intervals (Morey, 2008)

Regarding the GLM-based analysis, the rmANOVA on iRTs revealed the statistical significance of all the investigated effects. The post-hoc comparisons on the main effects of Task [*F*(3.44, 244.31) = 33.9, *p* < .0001, *η*^2^_*p*_ = .32] revealed that participants were significantly slower in performing the Peripheral task as compared to all the other tasks (all *p*s < .003) and significantly faster in performing the Saliency task as compared to all the other tasks (all *p*s < .001). On the other hand, the Perifoveal, the Navon, the Figure-Ground and the Flanker tasks did not significantly differ from each other in terms of iRTs (all *p*s > .109). The effect of Congruency was also significant [*F*(1, 71) = 907.2, *p* < .0001, *η*^2^_*p*_ = .93], with a very high overall Stroop effect (M = .269, SD = .076). Crucially, the Stroop effects differed across tasks [*F*(3.33, 236.55) = 55.7, *p* < .0001, *η*^2^_*p*_ = .44], albeit they were all significant (all *p*s < .0001) with very high effect sizes (all *d*s > 1.57) and dominance values, that is, the percentage of participants showing a positive Stroop effect (see Fig. 2 and Table 2; see also osf.io/cwh73 for the detailed statistics; see also osf.io/49kdh and osf.io/ysmjc for the analyses on lnRTs and RTs, respectively). Indeed, the post-hoc comparisons on the follow-up ANOVA revealed the following pattern of Stroop effects: Perifoveal > Figure-Ground & Peripheral > Flanker > Navon & Saliency.

**Table 2:** Stroop effects for iRTs as a function of the analytical approach

	GLM					LMM					RCA				
	*M*	*SD*	*t*	*d*	*Dom*%	*M*	*SD*	*t*	*d*	*Dom*%	*M*	*SD*	*t*	*d*	*Dom*%
Peripheral	0.324	0.137	20.1	2.37	100	0.343	0.113	25.7	3.02	100	0.346	0.145	20.3	2.39	100
Perifoveal	0.391	0.141	23.5	2.77	100	0.415	0.129	27.3	3.21	100	0.408	0.153	22.7	2.67	100
Navon	0.188	0.096	16.7	1.96	95.8	0.197	0.070	24.0	2.83	100	0.197	0.098	17.0	2.00	95.8
Figure**-**Ground	0.307	0.120	21.7	2.56	100	0.320	0.100	27.2	3.21	100	0.317	0.121	22.3	2.63	100
Flanker	0.252	0.120	17.7	2.09	98.6	0.262	0.104	21.4	2.53	98.6	0.259	0.119	18.4	2.17	98.6
Saliency	0.152	0.097	13.4	1.58	91.7	0.157	0.064	20.8	2.46	100	0.153	0.099	13.1	1.54	91.7

iRT, inverse-transformed RTs; GLM, general linear model; LMM, linear mixed-effects model; RCA, random coefficient analysis; *Dom*%, percentage of participants showing a positive Stroop effect (see Data Analysis section for details)

The ANOVA results were replicated by both multilevel modeling analyses. Indeed, the LMM analysis confirmed the statistical significance of the Congruency by Task interaction (*p* < .0001, see Table 3), with a similar pattern of the across-tasks differences in Stroop effects described above (see osf.io/djszu, Section 5.1.3). Moreover, albeit the pattern of participants’ Stroop effects was accurately recovered by LMM, with high correlations with the Stroop effects observed in the GLM analyses (all *r*s > .81), the corresponding effect sizes were all considerably larger, especially for the Peripheral and Perifoveal tasks, which resulted significantly larger (see Table 2; see also osf.io/djszu, Sections 3 and 5.1.4). This was likely due to the partial pooling (also called shrinkage or regularization) used by LMMs to estimate random slopes, which reduced the between-subject variability of the Stroop effects by “shrinking” the participant-specific effects toward the sample average effect based on the assumed normality of their distribution. Consequently, the LMM consistently underestimated the Stroop effects of participants showing larger Stroop effects and overestimated those of participants showing smaller Stroop effects. Indeed, linear regression analyses revealed that, for all tasks, the slope of the regression line for the Stroop effects yielded by the LMM relative to the GLM approach was significantly lower than 1, while the intercept was significantly larger than 0 (see osf.io/djszu, Section 5.1.4).

**Table 3:** LMM results

Effect	*F*	DF1	DF2	*p*
Block	34.5	1	192.70	< .0001
Trial	729.4	1	28887	< .0001
postERR	437.3	1	29124	< .0001
preRT	799.3	1	28950	< .0001
hResp	111.9	1	28862	< .0001
vResp	375.7	1	28859	< .0001
Trial:Block	23.2	1	28868	< .0001
CONG	437.3	1	72.23	< .0001
TASK	51.6	5	71.21	< .0001
CONG:TASK	58.2	5	88.46	< .0001

postERR, post-error trials; preRT, iRT at the previous trial; hResp, horizontal coding of the response (i.e., the responding hand: right vs left); vResp, vertical coding of the response (i.e., the responding finger: middle vs index); CONG, Congruency; DF, degrees of freedom. *P* values were computed using the Satterthwaite’s approximation

The LMM analysis also revealed that all the confounding predictors significantly modulated participants’ iRTs (all *p*s < .0001, see Table 3). Specifically, the Trial by Block interaction indicates a learning/adaptation effect (i.e., a reduction of iRTs as the trials within a block went on) that decreased during the experiment (i.e., as the blocks went on). Moreover, there was a significant post-error slowing (i.e., participants’ iRTs were significantly higher after an erroneous trial) and a significant temporal dependency in iRTs (i.e., a positive correlation between iRTs at the current and preceding trial). Finally, participants were faster on average in providing a response with the index fingers and with the right hand (note that the participants’ EHI score did not significantly modulate this latter effect: *χ*^2^(2) = 0.81, *p* = .668).

The conditional *R*^2^ of the LMM model was .70 and 0.99% of the observations was removed as outliers (>3 absolute standardized residuals) to mitigate the stress of the model fit (i.e., to improve the normality of the residuals, see osf.io/djszu, Section 5.1.2). The same model was also fitted on both RTs and lnRTs, confirming the results reported above (see osf.io/djszu, Sections 5.2 and 5.3). As anticipated above, however, the assumptions of homoscedasticity and normality of the residuals were violated in the analyses on lnRTs and RTs (see osf.io/djszu, Sections 5.2.2 and 5.3.2), so results obtained on these data should be taken with caution. Moreover, it should be noted that the inclusion of the by-participants random slopes for the Congruency by Task interaction of interest resulted in a singular fit in all cases, likely due to the limited number of observations.

The control LMM analysis performed using the reduced model without confounding predictors confirmed the across-task pattern of Stroop effects, with very high correlations with the Stroop effects observed using the full model (all *r*s > .983). However, participants’ Stroop effects obtained using the reduced model were all significantly and consistently smaller than those obtained in the main LMM analysis controlling for the trial-level confounding effects (all *p*s > .0001; see osf.io/djszu, Section 5.4).

We thus performed the RCA analysis, which replicated the LMM results (see osf.io/djszu, Section 6.1.1). Indeed, the Congruency by Task interaction was significant, with the same pattern of across-tasks differences in Stroop effects revealed by the GLM analysis (see above, see also osf.io/djszu, Section 6.1.3). Notably, both the raw and standardized effect sizes for the Stroop effects were almost identical to those revealed by the GLM analysis (see Table 2). Indeed, the pattern of participants’ Stroop effects was recovered almost perfectly by RCA, as the correlations with the Stroop effects observed in the GLM analyses were all very high (all *r*s > .96, see osf.io/djszu, Sections 3 and 6.1.4) and higher than those observed for the LMM analysis in all tasks (see osf.io/djszu, Section 6.1.5).

Moreover, all the effects of the confounding predictors on participants’ iRTs were confirmed (all *p*s < .001; see osf.io/djszu, Section 6.1.1, for the detailed statistics). The conditional *R*^2^ of the RCA model was .71 and 0.8% of the observations was removed as outliers to mitigate the stress of the model fit (see osf.io/djszu, Section 6.1.2). Again, the same model was also fitted on both RTs and lnRTs, confirming the results reported above (see osf.io/djszu, Sections 6.2 and 6.3), but with violations of the assumptions of homoscedasticity and normality of the residuals (see osf.io/djszu, Sections 6.2.2 and 6.3.2). To further check the robustness of our results, we ran similar RCA analyses after excluding the postERR predictor. Indeed, given the high accuracy, some participants had very few post-error trials, making the estimation of the post-error slowing effect problematic. These analyses confirmed the results reported above (see osf.io/9xfkw).

The control RCA analysis performed using the reduced model without confounding predictors confirmed the across-task pattern of Stroop effects, with very high correlations with the Stroop effects observed using the full model (all *r*s > .976). However, as for the LMM analysis, participants’ Stroop effects obtained using the reduced model were all significantly and consistently smaller than those obtained in the main RCA analysis controlling for the trial-level confounding effects (all *p*s > .025; see osf.io/djszu, Section 6.4).

Finally, the correlational analysis performed to examine commonality across spatial Stroop tasks revealed a specific pattern of intercorrelations among Stroop effects that emerged in all the three analyses we performed (see osf.io/djszu, Sections 7.1-3). Specifically, there was a significant correlation between the Stroop effects yielded by the Peripheral and Perifoveal tasks (*r* = .627, .860, and .594 for the GLM, LMM, and RCA analyses, respectively). Moreover, the Stroop effects for the Saliency task was significantly correlated with those yielded by the Navon (*r* = .304, .472, and .296), Figure-Ground (*r* = .491, .860, and .485), and Flanker (*r* = .443, .802, and .425) tasks. Finally, the Stroop effects yielded by the Figure-Ground and Flanker tasks were significantly correlated (*r* = .720, .878, and .720). The exploratory factor analysis confirmed this pattern of results. Indeed, it revealed the existence of two latent factors that consistently comprised, respectively, the Peripheral and Perifoveal Stroop effects (all loadings > .73) and the Figure-Ground, Flanker, and Saliency Stroop effects (all loadings > .53). The Navon Stroop effect was inconsistently included in the first factor with small loadings (< .44) and high uniqueness (> .70) (see osf.io/djszu, Section 7.4; see also osf.io/zqemg).

### Internal reliability of the Stroop effects

**Fig. 3:**
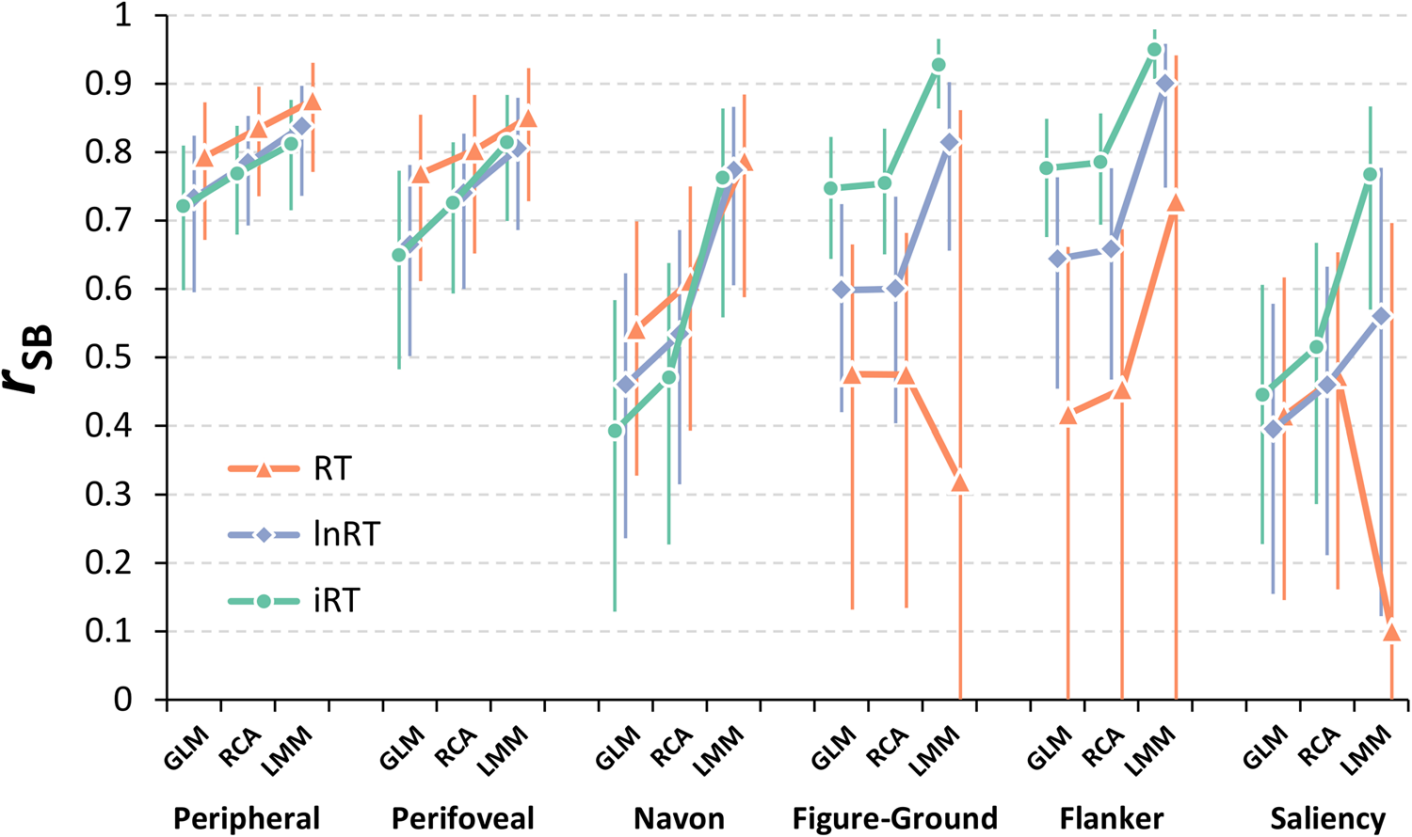
Internal reliability of the Stroop effects. The plot shows the median internal reliability estimates (*r*_SB_) of the Stroop effects for each task as a function of both the analytical approach (GLM: general linear model, RCA: random coefficient analysis, LMM: linear mixed-effect model; *x-axis*) and RT transformation (RT: untransformed response times –*in orange*–, lnRT: natural log-transformed RT –*in purple*–, iRT: inverse-transformed RT –*in green*–; see Data analysis section for details). The *error bars* represent the nonparametric 95% confidence interval

Figure 3 shows the internal reliability estimates (the median *r*_*SB*_ and the corresponding nonparametric *CI*_95%_, see also osf.io/djszu, Section 8) of the Stroop effects for each task as a function of both the analytical approach and the RT transformation.

A first notable result regards the influence of the analytical approach on the internal reliability estimates, which generally tended to be higher and more stable for RCA and LMM approaches as compared to the GLM one. By contrast, the RT transformations differently affected the internal reliability estimates across versions.

Nonetheless, the internal reliability of both the Peripheral and Perifoveal versions was quite high and robust against the choice of the analytical approach and the RT transformation. Indeed, the median *r*_*SB*_ values for these versions were consistently higher than .65 (range: from .72 to .88 and from .66 to .85, respectively) with relatively low variability across randomizations, albeit they still tended to be higher and more stable for RCA and LMM approaches as compared to GLM one, and lower for lnRTs and iRTs as compared to untransformed RTs.

By contrast, the internal reliability of the other versions was generally lower and less robust as compared to that of the Peripheral and Perifoveal versions, with higher variability of median *r*_*SB*_ values not only across randomizations, but also as a function of both the analytical approach and the RT transformation. Specifically, the internal reliability of the Navon version ranged from .39 to .79, with a similar pattern as the one observed for the Peripheral and Perifoveal versions. Conversely, the internal reliability of both the Figure-Ground and Flanker versions (median *r*_*SB*_ values range: from .32 to .93 and from .42 to .95, respectively) tended to be higher and more stable for lnRTs and, especially, iRTs as compared to RTs. Particularly good internal reliability estimates were observed for both versions in the LMM analyses on iRTs. Finally, the internal reliability of the Saliency version was generally poor (median *r*_*SB*_ values range: from .1 to .77), again except for the one observed in the LMM analyses on iRTs.

Interestingly, the internal reliability of the Stroop effects yielded by the control multilevel analyses performed using the reduced models (i.e., without including the trial-level confounding predictors) was lower than that obtained using the full models (see osf.io/djszu, Table S56), which also yielded larger Stroop effects.

## Discussion

The spatial Stroop task is an experimental paradigm measuring cognitive control and interference resolution that offers some methodological advantages over the commonly used verbal Stroop task. However, the spatial Stroop tasks used in the literature all have methodological drawbacks that limit their potential of yielding a complete Stroop interference effect that is analog to that engendered by verbal Stroop tasks. We recently proposed a spatial Stroop task (Puccioni & Vallesi, 2012a) that overcomes these limitations by ensuring conflict at multiple loci, that is, at the task, stimulus and response levels, with complete alternated trials sequences, thus allowing to measure participants’ interference resolution and conflict adaptation abilities effectively. Nonetheless, this task (Peripheral) still has some weaknesses due to the use of peripherally presented visual stimuli. Therefore, we aimed at assessing alternative spatial Stroop versions that maintain the methodological advantages of the Peripheral spatial Stroop task while overcoming its limits. To this aim, we designed five novel versions of the spatial Stroop task (Perifoveal, Navon, Figure-Ground, Flanker, and Saliency) and performed an online study to compare the six spatial Stroop tasks in a within-subjects design. Although we predicted that they all would have produced a spatial Stroop effect, our goal was to identify the task yielding not only the largest and most dominant, but also the most reliable Stroop effect. Moreover, we performed various analyses to assess the robustness of our results to analytic flexibility.

## Magnitude of the Stroop effects

The analyses assessing the magnitude of the Stroop effects in our tasks revealed that they all yielded very large Stroop effects. Indeed, regardless of the RT transformation or the analytical approach, the d values for all tasks were always well above the value of 0.8 (see Table 2), which is commonly considered as the threshold of large effect sizes. This confirms our expectation that all our spatial Stroop paradigms are effective at producing a spatial Stroop effect, in line with the dominance assumption (Rouder & Haaf, 2018, 2019). Our results are thus generally in line with the idea that all individuals have a true positive Stroop effect (i.e., they truly respond more slowly in incongruent compared to congruent trials).

In particular, our original Peripheral Stroop task seems to be universal (Parsons, 2020), with all participants showing an estimated positive Stroop effect that was very large and robust to analytic flexibility (median *d* = 2.09; range: from 1.43 to 3.02; see osf.io/djszu, Section 3), with the exception of one participant in the RCA analysis on RTs (who nonetheless showed an estimated raw Stroop effect of –1.7 ms, which was indistinguishable from a positive Stroop effect with the resolution of our experimental design). This result confirms our previous findings showing the complete dominance of the Stroop effect yielded by the Peripheral task: all the 287 and 57 participants in the Capizzi et al.’ (2017) and the Ambrosini and Vallesi (2017) studies, respectively, showed an estimated positive Stroop effect in a GLM-based analysis on lnRTs. Our analyses on the iRTs also revealed that this task yielded a Stroop effect that was consistently larger than those observed in the Navon, Flanker, and Saliency tasks (see osf.io/djszu, Section 3).

Similar results were obtained for the Perifoveal task, which is a variation of the Peripheral task. Indeed, also in this case all participants showed an estimated positive Stroop effect that was robust and even larger than that yielded by the Peripheral task (median *d* = 2.47; range: from 1.65 to 3.23; see osf.io/djszu, Section 3), again except for one participant in the RCA analysis on RTs (who showed a raw Stroop effect of –1.8 ms). Specifically, all the analyses on iRTs revealed that the Perifoveal Stroop effect was significantly larger as compared to that observed for all the other tasks, including the original Peripheral one (see osf.io/djszu, Sections 4.1, 5.1.3, and 1.1.3). Since the only difference between these two tasks is the spatial arrangements of the visual stimuli (and the consequently lower eccentricity), these results indicate that large visuospatial attention shifts and/or eye movements were indeed present in the Peripheral task and that they may have led to an underestimation of the corresponding Stroop effect. Some support for the former conclusion comes from the significant main effect of Task, which showed that the Peripheral task had the longest overall RTs, confirming our initial assumption of its methodological limitations. Notwithstanding this difference, our results clearly indicated the commonality between the Peripheral and Perifoveal tasks, as their iRT Stroop effects consistently showed a significant correlation (≥ .60) and loaded on the same latent factor, suggesting that they were related to the same cognitive processes (see osf.io/djszu, Section 7).

Our analyses also revealed that the universality assumption holds for the Figure-Ground task as well, albeit it presents more radical methodological variations as compared to the two tasks discussed above. In this case, all participants showed an estimated positive Stroop effect that was robust to analytical flexibility and very large (median *d* = 3.02; range: from 2.56 to 5.52; see osf.io/djszu, Section 3). Specifically, all the analyses on iRTs revealed that the Figure-Ground Stroop effect was comparable to that observed in the Peripheral task and significantly larger as compared to those observed in the Navon, Flanker, and Saliency tasks (see osf.io/djszu, Sections 4.1, 5.1.3, 6.1.3).

Finally, the Flanker task showed very large Stroop effects (median *d* = 2.49; range: from 2.07 to 4.76) but with a lower level of dominance because some participants did not show a positive Stroop effect. By contrast, the Navon and, especially, the Saliency tasks yielded Stroop effects that were smaller and less robust than those observed for the other tasks (see osf.io/djszu, Section 3).

### Internal reliability of the Stroop effects

Despite the dominance and large magnitude of the Stroop effects yielded by the Peripheral and Perifoveal tasks, their internal reliability was quite high and robust against the choice of the analytical approach and the RT transformation, also with a relatively low variability across randomizations (see Fig. 3 and osf.io/djszu, Section 8). This is at odds with the reliability paradox (Hedge et al., 2018a) and related proposals (Rouder & Haaf, 2019) claiming that if an experimental effect is so large and easily replicable to be called universal (Parsons, 2020), like the ones we obtained here, it would likely tend to have a between-subjects variability that is not large enough to ensure an adequate reliability^3^. Although the reliability of the spatial Stroop effects has hardly ever been reported, our results are in line with Paap et al. (2020), who reported a split-half correlation of .81, and with a recent large individual difference study (*n* = 287, Capizzi et al., 2017) in which we used our Peripheral task to detect a very large spatial Stroop effect on lnRTs (*d* = 2.96) with an adequate internal reliability (*r*_SB_ = .77). It is also important to note that, given the limited number of trials we used, the internal reliability estimates we obtained here likely represent a lower bound on the actual internal reliability of these tasks. Indeed, as also shown by Hedge and coworkers (2018a), there is a clear positive nonlinear relationship between reliability and number of trials. In other words, there is room for improvement for our Peripheral and Perifoveal spatial Stroop task as, by increasing the number of trials, their reliability will inevitably increase.

Concerning the reliability of the Stroop effects yielded by the other tasks, it was quite high for the Figure-Ground task - despite the dominance and large magnitude of the Stroop effects -, at least for the analyses on iRTs. A similar pattern of results was obtained also for the Flanker task, which showed a good internal reliability, despite very large Stroop effects. On the contrary, the Navon and the Saliency tasks, which showed smaller Stroop effects, were also less reliable (see Fig. 3 and osf.io/djszu, Section 8).

### Impact of the analytical approach

Our results provide support for the idea that multilevel modelling can improve reliability estimates of the experimental effects by separating true experimental effects from measurement error (i.e., the intra-subject, trial-by-trial variability, also called trial noise). Indeed, both the effect size and, especially, the reliability of our iRT Stroop effects were consistently higher for the LMM and RCA analyses as compared to the GLM analysis, which requires aggregating participants’ measures across trials, thus ignoring any trial-by-trial variability that may contaminate them and, thus, estimates of experimental effects. More importantly, they were also consistently higher than those estimated using the reduced LMM and RCA models that did not include the confounding predictors accounting for (at least part of) the measurement error. Following the “reliability paradox” study (Hedge et al., 2018a), some attempts using multilevel modelling have been made in trying to improve reliability estimates of experimental effects and their correlations (Haines et al., 2020; Rouder & Haaf, 2019), with some promising results, albeit it has been shown that the use of more complex analytical approaches of this type is not sufficient alone to uncover the correlations between experimental effects (Rouder & Haaf, 2019).

Here we took a step further by using the multilevel modelling approach in a more informed way, not only to simply remove trial noise, but also to explain and partial out the effects of lower-level confounding factors at the trial level. Indeed, we claim that part of the intra-subject, trial-by-trial variability is not just noise: at least in part, it represents the effects of well-known longitudinal effects and other confounding effects due to other perceptual, motor, and cognitive processes. These effects can be assumed based on the specific characteristics of the task at hand and the psychological mechanisms that generate behavior. Therefore, they can be included in the analytical model to improve the estimates of the experimental effects of interest and of their reliability as well. Of course, as we detailed in the Introduction, some of these effects, like the sequential effects due to the trial-by-trial repetitions of the stimulus characteristics, can (and should, whenever possible) be controlled methodologically. We thus advocate the use of such a theoretically-informed methodological approach in creating (or selecting) experimental tasks, followed by an informed hierarchical analytical approach to estimate the corresponding experimental effects.

### Differences across the task versions

Apart from the evident communality between the Peripheral and Perifoveal tasks that we discussed above, our correlational and exploratory factor analyses were also able to identify the specific methodological differences among our tasks that are important to note here (see osf.io/djszu, Section 7). First, in the Peripheral and Perifoveal tasks, the irrelevant characteristic of the stimulus is intrinsic to the stimulus itself, while in the Figure-Ground, Flanker, and Saliency tasks it constitutes an extrinsic dimension, that is, it is a physically separate stimulus. The Navon task is peculiar in this aspect because the relevant stimulus (i.e., the large arrow) is created by illusory contour perception given by the spatial arrangement of the irrelevant stimuli (i.e., the small arrows).

Moreover, in the Peripheral and Perifoveal tasks the relevant and irrelevant characteristics (i.e., the position and direction of the arrow, respectively) are different dimensions that are related to different perceptual processes (i.e., the spatial localization of a visual stimulus and the perception of its shape), while in the other tasks the relevant and irrelevant stimulus dimensions not only overlap but are identical and related to the same perceptual process, constituting what Kornblum called a “super-Stroop ensemble” (1992, p. 770). The involvement of a single perceptual process in the Figure-Ground, Navon, Flanker and Saliency tasks might be questionable because it could be interpreted as the absence of task conflict, which, in contrast, has been shown to be required for a complete Stroop effect (Augustinova et al., 2019; Parris et al., 2022). Since we agree with the multiple-loci accounts of the Stroop effect and, recently, we have stressed the importance of methodologically correct spatial Stroop designs (Viviani et al., 2022), we need to clarify why these four tasks can be considered fully-fledged Stroop tasks, ensuring interference not only at the stimulus and response levels but also at the task level. As pointed out in the introduction, they are also characterized by an asymmetrical relation between the two dimensions but, as opposed to the Peripheral and Perifoveal tasks, such asymmetry does not only rely on higher processing automaticity but also on higher perceptual salience of one dimension as compared to the other. Therefore, task conflict is present because our task-relevant stimuli still differ from the task-irrelevant ones from a conceptual and/or perceptual point of view, and, consequently, they activate task sets that, despite belonging to the more general shape perception processing stream, are distinguishable. This is particularly striking in the Navon version, for which the distinction between local and global processing, together with global precedence, are well-documented. Therefore, it is highly likely that the prevailing but task-irrelevant global processing interferes with the less habitual but task-relevant local processing. This assumption can be extended to the Figure-Ground task, wherein the distinction between global vs local processing streams might be also influenced by perceptual factors. The competing task sets activated in the Flanker and in the Saliency tasks are instead more likely to be perceptual in nature. In the former, the task-irrelevant processing of surrounding arrows may be stronger because of their higher quantity as compared to the single and less salient central task-relevant arrow, whereas in the second, the task-irrelevant processing of the bigger arrow is stronger than the task-relevant processing of the smaller one for its greater size and saliency.

The pattern of the across-tasks correlations we observed and the results of the exploratory factor analysis could thus reflect these across-tasks methodological differences, which, in turn, could explain our results on the magnitude and reliability of the different Stroop effects. For example, taking the most evident difference concerning task conflict, the fact that the Stroop effect was the most robust and reliable in the Peripheral and Perifoveal tasks might indicate that in these tasks the degree of interference at the task level was the highest, probably due to the stronger asymmetry between position and direction processing. When, in contrast, such conflict was involved to a lesser extent due to a more perceptually based asymmetry, such as in the remaining four tasks, both Stroop effect magnitude and reliability were lower, albeit quite good, especially in the Figure-Ground and Flanker tasks. These findings provide further evidence for the multiplicity of Stroop effect loci, confirming the importance of including all the required interference levels when designing Stroop tasks.

Apart from the above-mentioned methodological differences related to interference resolution, the observed across-task differences in Stroop effect magnitude and reliability could also be explained, at least in part, by differences in non-conflict processes. For example, the across-task pattern of RT Stroop effects derived from the GLM analysis, reported in Table 1, seems to be related to the across-task pattern of RTs in Congruent trials: tasks with longer baseline RTs tended to produce larger Stroop effects. This could reflect something similar to what generally observed, for example, in studies contrasting behavioral performance in older vs. younger adults (Faust et al., 1999). Therefore, across-task differences in general processing demands (or task difficulty) or other non-conflict processes could have modulated the corresponding Stroop effects independently of the underlying differences in the produced interference that we discussed above. In line with this interpretation, recent simulations from evidence accumulation models produced larger RT Stroop effects with decreases in mean drift rates (reflecting general processing speed) and increases in boundary separation (reflecting strategic slowing – or more cautious responding) while keeping conflict effects constant (Hedge et al., 2018b, 2022). However, the relation between Stroop effect magnitude (and reliability) and baseline performance/task difficulty across our tasks was strongly dependent on the analytical choices. Indeed, it was less evident both in the multilevel analyses on RTs and for RT transformations (see osf.io/djszu, Fig. S3); Moreover, it was strongly driven by the results of the Peripheral task, which showed the slowest baseline performance and very large (and reliable) Stroop effects. More research is thus required to clarify the impact of task general processing requirements and other non-conflict processes on the magnitude and reliability of Stroop effects.

Overall, these results and our methodological considerations highlight the importance for the researchers interested in using the tasks presented here to carefully consider their specific characteristics in light of their research questions, as they may have a non-negligible impact on the ability to identify the behavioral and neurophysiological correlates of Stroop interference resolution. Moreover, albeit preliminary, our results suggest that our tasks were accurately tailored to adequately assess inter-individual variability in interference resolution and conflict adaptation in different populations^4^ and provide further support for the possibility to use these experimental effects in correlational research (e.g., see Ambrosini & Vallesi, 2017).

## Conclusions

Overall, our results suggest that the best alternative to our original Peripheral task is the Perifoveal task. Indeed, they both showed a Stroop effect that was so large and robust to analytical flexibility to be used as a measure of interference resolution and conflict adaptation that satisfies the dominance assumption. Moreover, both the Peripheral and Perifoveal tasks showed an adequate internal reliability, making them viable options for scholars interested in conducting correlational research. The Figure-Ground and the Flanker tasks also showed a good balance between the reliability and the magnitude of the Stroop effect, but they also showed less robustness to analytical flexibility.

## Data Availability

All data and materials are available from our project repository at the Open Science Framework here: osf.io/5sm9j/.

## Footnotes

1 When congruent trials are used as the baseline condition against which to compare performance in incongruent trials, the obtained experimental effect is usually called Stroop effect. Alternatively, neutral trials (e.g., a colored non-word string) can be used as baseline condition, allowing to partition the Stroop effect into Stroop interference and facilitation effects. Here we chose to focus on the Stroop effect because most of the existing studies (including those assessing reliability) investigated it rather than its subcomponents. Moreover, it is still not clear what type of neutral stimulus should be used to better estimate facilitation and interference effects. Finally, to the best of our knowledge, neutral trials were never used in the (methodologically correct) spatial versions of the Stroop task. For a detailed discussion of this issue, see our recent review (Viviani et al., 2022).

2 Stimulus-stimulus overlap does not need to be spatial, but, as long as the task-relevant and task-irrelevant stimuli are perceived as an ensemble, it can be conceptual too, as it was in our case, in which they are both presented in perifoveal vision.

3 There is indeed a selection pressure in experimental psychology for tasks showing robust and consistent effects, which could be obtained either by increasing the mean of their raw effect size or by decreasing its inter-individual variability. However, while the former strategy could improve the reliability of the task, as suggested by our results (see also Rouder, Kumar & Haaf, 2019), the latter is assumed to have the opposite effect. This issue is further complicated by the fact that there is a strong positive correlation between the mean and the variability of experimental effects, especially the universal ones that can be assumed to be bounded to be positive. However, our study does not allow us to tackle this issue with the required methodological rigor (due to, for example, the limited number of tasks, participants, and trials), so further studies are needed to draw sensible conclusions about it.

4 Note indeed that the difficulty level of the tasks, as reflected by the accuracy results we presented, makes them suitable to be employed in experimental studies on populations with lower performance level, like children, older participants, and even patients.

## Code availability

The code used to run the experimental tasks and generate and analyze the data of the current study is available from our project repository at the Open Science Framework here: osf.io/5sm9j/.
